# Vitamin D in the Treatment of Oral Lichen Planus: A Systematic Review

**DOI:** 10.3390/biomedicines10112964

**Published:** 2022-11-17

**Authors:** Shazina Saeed, Priyadarshini Choudhury, Syed Ansar Ahmad, Tanveer Alam, Rajat Panigrahi, Shahid Aziz, Sultan Mohammed Kaleem, Smita R. Priyadarshini, Pradyumna Ku Sahoo, Shamimul Hasan

**Affiliations:** 1 Amity Institute of Public Health, Amity University, Noida 201303, India; 2 Nahar Hospital, Bhinmal 343029, India; 3 Department of Oral and Maxillofacial Surgery, Faculty of Dentistry, Jamia Millia Islamia, New Delhi 110025, India; 4 Department of DDS, College of Dentistry, King Khalid University, Abha 61413, Saudi Arabia; 5 Department of Oral Medicine and Radiology, Institute of Dental Sciences, Siksha ‘O’ Anusandhan University, Bhubaneswar 751003, India; 6 Department of Medicine, College of Medicine, King Khalid University, Abha 61413, Saudi Arabia; 7 Department of Prosthodontics, Institute of Dental Sciences, Siksha ‘O’ Anusandhan University, Bhubaneswar 751003, India; 8 Department of Oral Medicine and Radiology, Faculty of Dentistry, Jamia Millia Islamia, New Delhi 110025, India

**Keywords:** autoimmune, dentistry, oral lichen planus, oral pathology, treatment, vitamin D

## Abstract

Oral lichen planus (OLP) is a chronic mucocutaneous condition that affects up to 2% of the general population, and typically presents with long-standing, non-responsive lesions, with episodes of exacerbation and remissions. The etiopathogenesis of OLP is still unclear, although, it has been postulated that it is most likely a T-cell-mediated condition of an unknown antigen. The treatment remains a challenge with no defined treatment strategy. Vitamin D has anti-inflammatory and immunomodulatory properties, along with its regulatory effect on keratinocyte proliferation and differentiation; thus, suggesting its possible role in the treatment of OLP. This systematic review aims to evaluate the therapeutic role of vitamin D in OLP treatment. We searched PubMed/MEDLINE, and Google scholar search engines for studies evaluating vitamin D as a treatment modality in OLP from January 2000 to August 2022. Articles were searched with the combination of Medical Subject Heading (MeSH) terms. A web platform for visualizing risk-of-bias assessment was used in this review, and descriptive statistics were calculated. Out of the seventeen retrieved studies, five articles meeting the inclusion criteria were considered in this systematic review. All the included studies demonstrated significant amelioration in the OLP symptoms in patients who were given vitamin D supplements as an adjuvant to the conventional steroid therapy and or placebo. This systematic review signifies the role of vitamin D as adjuvant therapy for OLP. However, more studies with larger sample size are required to validate these results.

## 1. Introduction

The term lichen planus (LP) was initially described by Erasmus Wilson (1869) as a chronic inflammatory, autoimmune ailment, primarily affecting the skin, oral and genital mucosa, and with a potential for undergoing malignant alterations. LP may also affect the hair follicles (lichen planopilaris, causing scarring alopecia), and nail appendages (nail ridging and pterygium formation), with an infrequent affiliation for the ocular, nasal, and laryngeal mucosa [1].

Oral involvement is a common occurrence, and in 15–35% of cases, oral mucosa may be the only affected site of the disease [2]. Oral lichen planus (OLP) represents the mucosal counterpart of the cutaneous LP [3] and typically presents with episodes of exacerbation and remission [4]. According to a recent systematic review and meta-analysis, the overall prevalence of OLP in the general population is 0.89% and 0.98% among clinical patients and varies according to geographic location. OLP has an age and gender predilection, primarily affecting females over 40 years of age [5].

Generally, the cutaneous LP lesions are self-limiting and occasionally pruritic, in contrast to the long-standing, and non-responsive oral lesions. The malignant potential of oral lesions frequently attributes to the associated morbidity [4,6].

OLP is currently considered an oral potentially malignant disorder (OPMD), although its malignant transformation rate is controversial, largely attributable to the restrictive criteria for its diagnosis (due to the use of varied inclusion and exclusion criteria in previous follow-up studies) [7].

Recent studies have reported that the malignant transformation rate of OLP ranges from 0.44% to 2.28% [8,9,10,11,12]. However, there is an increased risk of malignant potential in cases of erosive and/or atrophic lesions, tongue lesions, greater intake of alcohol/tobacco, and an accompanying hepatitis C virus infection [8,11,12,13].

OLP lesions characteristically manifest as bilaterally symmetrical reticular lesions on the buccal mucosa, tongue, and gingiva, although, involvement of the palatal mucosa, lips, and floor of the mouth is infrequently seen [14].

OLP may manifest a plethora of clinical forms, and range from reticular, erosive, atrophic, plaque-like, papular, and bullous lesions [1,4,15]. Generally, reticular lesions are the commonest, and the bullous/papular forms are the rarest oral presentations [1,4]. The most common reticular form of OLP is asymptomatic, whereas, the atrophic, erosive, and bullous forms usually cause pain, burning sensations, difficulty in mastication and speech, and deteriorated oral hygiene. These forms are also associated with negative psychosocial outcomes due to the chronic, uncertain clinical patterns and potential for malignant transformation, thus affecting the patient’s quality of life [2,16].

Despite breakthrough research and substantial knowledge advancements, the etiopathogenesis of OLP is still ambiguous, and OLP is regarded as a chronic T-cell-mediated disorder of unknown etiology [14]. However, a plethora of multifactorial predisposing factors, such as autoimmunity, microorganisms, infective agents, drugs and dental materials, nutritional deficiencies, psychological stress, and genetic predisposition may also have a role to play [4,14,17]. The ambiguous nature of T lymphocytes is considered to be the trigger factor that may predispose oral mucosa to undergo apoptosis involving auto-cytotoxic T-lymphocytes [3].

OLP is a very debatable condition due to the absence of specific diagnostic criteria. WHO diagnostic criteria were modified by Van der Meiji and van der Waal (2003), in which the absence of epithelial dysplasia confirmed OLP diagnosis, thus, attempting to exclude lichenoid dysplasia from OLP [18]. The American Academy of Oral and Maxillofacial Pathology (2016) recommended diagnostic criteria for OLP, asserting clinicopathologic corroborations to establish the diagnosis [14]. A recent systematic review and meta-analysis strengthened the incorporation of clinical and histopathological features for definitive OLP diagnosis. Comprehensive documentation incorporating all demographic, medical, and environmental variables is imperative for OLP cases [8].

Treatment strategies are focused on precluding the excruciating symptoms, hastening the remission of erosive lesions, enhancing the asymptomatic periods, diminishing the malignant transformation risk, and maintaining good oral hygiene and dental status [19,20]. However, there is no conclusive therapeutic regimen for OLP due to its obscure etiopathogenesis and recalcitrant nature [21]. Reticular OLP is usually asymptomatic and does not require any treatment. Regular follow-up and assessment are generally preferred for these lesions [4,19].

Several pharmacological and non-pharmacological treatment regimens have been advocated for the management of OLP. The pharmacological therapeutic modalities used in the treatment of OLP include corticosteroids (topical, intralesional, and systemic steroids), Immunosuppressants (tacrolimus, azathioprine, cyclosporin, and mycophenolate mofetil,), immunomodulators (levamisole and thalidomide), and retinoids. Various non-pharmacological regimens, such as Light amplification by stimulated emission of radiation (LASER) therapy, photodynamic therapy, and Psoralen plus ultraviolet-A radiation (PUVA) therapy are also used in OLP treatment [19,22]. A range of alternative therapies, such as topical aloe vera, oral curcuminoids, lycopene, hyaluronic acid, Bacillus Calmette-Guerin Polysaccharide nucleic acid (BCG-PSN), purslane extract, and ignatia have also exhibited promising results in the management of OLP [23].

A recently published meta-analysis conducted on 55 randomized controlled trials evaluated several OLP treatment protocols and considered topical corticosteroids to be the most efficacious treatment modality [24]. Systemic steroids are allocated for cases where topical therapies were ineffective, refractory erythematous/erosive lesions, or diffuse OLP lesions with accompanying cutaneous, scalp, and genital lesions [25].

Several different preparations and classes of topical steroids exist, that vary in efficacy and cost. Various local and systemic adverse effects may limit extended steroid use. Moreover, not all patients have a favorable response to steroids. All these factors necessitate an alternative treatment protocol for OLP [26].

Vitamin D belongs to the group of fat-soluble secosteroid biomolecules, procured in the body chiefly through the endogenous synthesis in the skin under UV radiation, and also in the diet (food products and food additives intake) [27].

Once obtained in the body, vitamin D undergoes modifications to become a biologically active metabolite (calcitriol). Vitamin D is converted to 25-hydroxyvitamin D (25(OH)D)/calcidiol by the enzyme 25-hydroxylase in the liver. Later, the enzyme 1α-hydroxylase further hydroxylates calcidiol to 1,25-dihydroxy vitamin D (1,25(OH)2D)/calcitriol in the kidneys [28].

Vitamin D is a pleiotropic hormone that primarily regulates serum calcium and phosphorus metabolism [29]. It also exhibits anti-proliferative, anti-angiogenic, pro-differentiating, and pro-apoptotic activities. A distinctive nuclear hormone receptor, the vitamin D receptor (VDR), is chiefly accountable for its biological actions [30]. Vitamin D regulates the cutaneous immune system homeostasis, differentiation and proliferation of keratinocytes, and apoptotic mechanisms. Vitamin D also exhibits anti-inflammatory actions and also modulates the adaptive and innate immune response [31,32].

Although the exact role of vitamin D in autoimmune disorders is still not distinctly delineated, vitamin D deficiency has been demonstrated in some autoimmune disorders such as type I diabetes mellitus, rheumatoid arthritis, systemic lupus erythematosus, inflammatory bowel diseases, multiple sclerosis, autoimmune gastritis, and autoimmune thyroid disorders (such as Graves’ disease and Hashimoto thyroiditis) [33].

The published literature has highlighted the role of vitamin D in several autoimmune mucocutaneous disorders. El-Komy et al. demonstrated significantly low serum vitamin D levels in patients with pemphigus Vulgaris. Vitamin D deficiency might serve as a predisposing factor and aggravate the disease through various immune-related mechanisms [34] Similar findings were also observed in a North Indian study on pemphigus Vulgaris patients [35]. The plausible role of vitamin D deficiency in the pathogenesis of bullous autoimmune mucocutaneous disorders and pemphigus Vulgaris has also been demonstrated in studies by Marzano et al. [36,37].

Considering the anti-inflammatory, and immunomodulatory properties of vitamin D, together with its regulatory effect on keratinocyte proliferation and differentiation, and the possible immune-mediated etiopathogenesis of lichen planus, vitamin D and its derivatives may be employed as a safe and efficacious treatment protocol for lichen planus [38]. Furthermore, an altered cytokeratin profile, possibly by intercellular complement and T-cell activation, thus inducing the inflammatory cascade has also been demonstrated in lichen planus. Vitamin D/analogs may facilitate the restoration of the normal epidermal cytokeratin profile, thus, further attributing to its therapeutic potential in lichen planus [38].

Few studies have ascertained the efficacy of topical calcipotriol (vitamin D3 analog) as a therapeutic protocol for cutaneous lichen planus [38,39]. However, there is a dearth of published literature assessing the therapeutic efficacy of vitamin D in OLP, with few published case reports [40,41].

Hence, this systematic review was carried out to establish and corroborate the therapeutic role of vitamin D in OLP. The review will also address the knowledge gaps which may pave the way to formulate new treatment guidelines for OLP patients.

## 2. Materials and Methods

This systematic literature review followed the PRISMA (Preferred Reporting Items for Systematic literature reviews and Meta-Analyses) 2020 guideline has been registered with PROSPERO (Registration ID-364794).

### 2.1. Research Question

The search for the systematic review was taken up by defining the keywords related to the population, intervention, control, and outcomes (PICO) format: (a) population– “oral lichen planus (OLP)”; (b) intervention/exposure– “Vitamin D therapy”; (c) control– “Healthy subjects, placebo or other interventions like corticosteroids, psychologic counseling”; and (d) outcome– “efficacy evaluation”.

Our focused research question was “to evaluate the therapeutic role of Vitamin D in patients with OLP”?

### 2.2. Inclusion Criteria

(a) Studies conducted on human subjects with OLP with Vitamin D supplementation; (b) articles published in the English language between January 2000 to August 2022; (c) sample size of a minimum of 10 study participants (both cases and control groups) (d) studies providing information on the therapeutic efficacy as an outcome; evaluated or measured by different methods of improvement (e.g., different objective and subjective clinical scales/systems).

### 2.3. Exclusion Criteria

(a) Studies evaluating the vitamin D serum levels in OLP patients but without vitamin D supplementation; (b) studies assessing the association of vitamin D receptors (VDRs) and OLP gene polymorphisms; (c) studies demonstrating the pathogenic pathways of OLP lesions due to vitamin D/VDR deficiency (d) studies conducted on human subjects with cutaneous lichen planus; (e) articles published in languages other than English and before January 2000; (f) study subjects less than 10; (g) subjects with underlying systemic disorders (h) case reports

### 2.4. Literature Search and Identification of Studies

The Preferred Reporting Items for Systematic reviews and Meta-Analyses (PRISMA) 2020 guidelines were used to design the methodology for this systematic review. The PRISMA statement includes a 27-item checklist that assures transparency, iteration, and complete reporting for systematic reviews. A detailed literature search on the PubMed/MEDLINE and Google Scholar databases was performed for observational studies evaluating vitamin D as a treatment modality in OLP patients from January 2000 to August 2022 using the following Medical Subject Headings (MeSH) terms, “Oral lichen planus”, AND “Vitamin D deficiency”, AND “Treatment”, OR “Therapeutics”. The search protocol was as follows: (“Lichen Planus, Oral” (Mesh) OR “Lichen Planus, Oral/drug therapy” (Mesh) AND “Vitamin D Deficiency/drug therapy” (Mesh) OR “Vitamin D Deficiency/therapeutic use “(Mesh) OR “Vitamin D Deficiency/therapy” (Mesh).

### 2.5. Study Selection

Two authors methodologically assessed the titles and abstracts of the retrieved studies, and any disparity was resolved by a third author. The full texts of the potentially eligible studies were then acquired and analyzed for further inclusion in the systematic review. The references of all the included studies were manually checked to include any previously missed studies.

### 2.6. Outcome Parameters

Various objective and subjective outcome scoring systems used by the included studies were appraised for evaluating the efficacy of various treatment protocols employed. Objective symptoms in the form of clinical appearance and severity of the lesions, and subjective symptoms in the form of pain and burning sensations evaluated on the visual analog scale (VAS) were considered.

### 2.7. Data Extraction

The following information was retrieved from the included articles: author name (s), publication year, the country where the study was conducted, study design, age and gender of the included subjects, sample size, the basis of OLP diagnosis, treatment protocol, the test of significance, and study outcome.

### 2.8. Risk of Bias Assessment

The risk of publication bias was assessed by using an R package and Shiny web app for visualizing risk-of-bias assessments introduced by the National Institute for Health Research (NIHR), as a part of the Doctoral Research Fellowship (DRF-2018-11-ST2-048) at the University of Bristol, UK. The current version from 2020 was used for the analysis. [42]. The program evaluates the following six domains: 1. randomization procedure, 2. recommended intervention, 3. missing outcome data, 4. assessment of the outcome, 5. selection of the outcome report, and 6. overall evaluation.

## 3. Results

**Figure 1:**
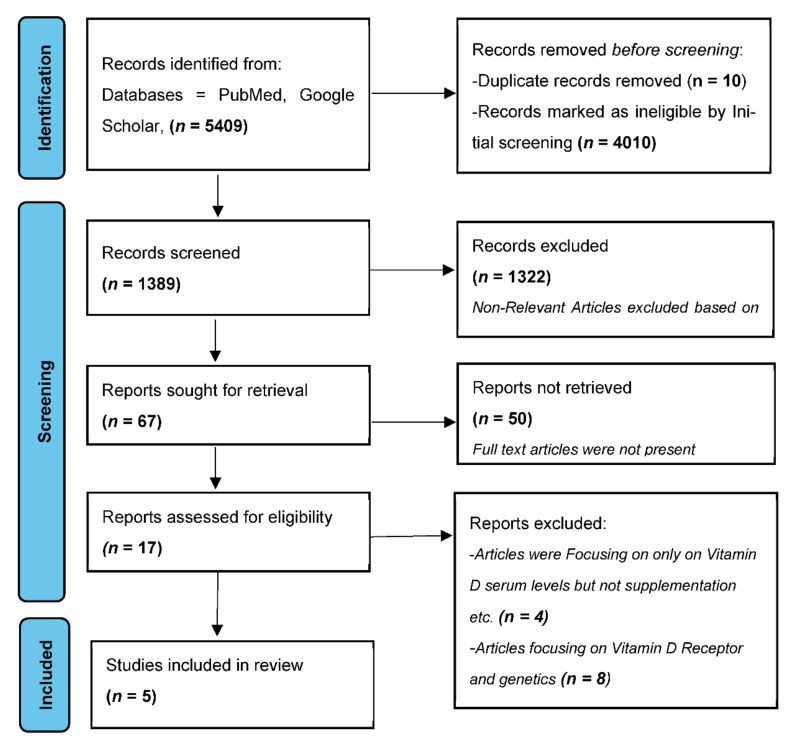
PRISMA flowchart showing the process of study selection.

Five articles were eventually considered eligible for inclusion and were further processed for data extraction [43,44,45,46,47]. Preferred Reporting Items for Systematic Reviews and Meta-Analyses (PRISMA) guidelines were followed for the literature search. The search strategy is illustrated as a flowchart in Figure 1.

### 3.1. Study Characteristics

A detailed description of the included studies is summarized in Table 1.

Out of the five studies (a total of 714 subjects) meeting the inclusion criteria, three were randomized controlled clinical trials [43,46,47], and two were observational studies [44,45]. Two studies were from India [44,45], and one study each was from Pakistan [43]. Egypt [46], and Iran [47]. Three studies included both genders [44,45,47], and two studies were conducted on females (one each on peri-menopausal and post-menopausal females) [43,46].

OLP was clinically diagnosed in two studies (based on bilateral, symmetrical interlacing Whickham’s striae, burning sensations, and intolerance to hot/spicy food) [43,46], whereas one study diagnosed OLP cases by the clinical and histopathological features based on World Health Organization (WHO) modified criteria [47]. However, two studies used the histopathological diagnosis only in doubtful cases (Gingival desquamation/inconspicuous bilateral symmetrical reticular pattern) [44,45].

Two studies compared the efficacy of standard OLP treatment with standard OLP treatment and vitamin D supplementation [43,46]. Two studies divided the subject population into three groups based on serum vitamin D levels and history of stress. Accordingly, the study groups were treated with topical steroids and psychological counseling, topical steroids, and vitamin D supplementation, and a combination of topical steroids, vitamin D supplementation, and psychological counseling, respectively [44,45]. One study evaluated the comparative efficacy of vitamin D supplementation and conventional steroid therapy with placebo and conventional steroid therapy [47].

### 3.2. Outcome Parameters

All five included studies evaluated the outcome based on the reduction in pain (VAS score) and difference in lesion size and appearance. The VAS score was in the range of 0 to 10, and a score between 0 to 4 was regarded as a significant improvement in pain intensity. The appearance and severity of the lesion were evaluated on a scoring system between 0 to 5: 0—no lesion/normal mucosa; 1—mild white striations/no erythematous area; 2—white striae with an atrophic area < 1 cm^2^; 3—white striae with an atrophic area > 1 cm^2^; 4—white striae with an ulcerative area < 1 cm^2^; 5—white striae with an ulcerative area > 1 cm^2^. After a gradual follow-up period, if a lesion was evaluated to be in the range of 0 to 2, it was considered a significant clinical amelioration of the lesion.

Razi et al. (2018) [43], was the first study to assess vitamin D supplementation as an adjunctive treatment modality of oral lichen planus among peri-menopausal women. A randomized control trial was carried out on peri-menopausal patients (age group of 35–45 years) with clinically diagnosed OLP and serum 25(OH) vitamin D levels below 30 ng/mL. The patients were divided into two groups: one group received conventional therapy (alternate use of two tablets betnesol 0.5 mg dissolved in 10 mL of benzydamine chlorhexidine mouthwash and four to five drops of Nilstat taken three times a day) and the other group received vitamin D supplementation along with conventional therapy. After a follow-up period at 1 week and 4 weeks, the pain intensity score of 5.04 ± 2.20 and 1.80 ± 0.40, *p*-value less than 0.001 was observed in group 1. Subjects in group 2 showed improvement in the clinical appearance of the lesion between week 1 (1.80 ± 0.40) and week 4 (0.80 ± 0.40), *p*-value less than 0.001. Patients in both groups experienced significant pain diminution. However, a marked amelioration in the clinical appearance of OLP lesions was observed in patients treated with vitamin D supplements and standard therapy (Table 1).

In an observational study by Gupta J et al. (2019) [44], OLP patients were divided into three different groups based on serum vitamin D levels and a history of stress, or a combination of both. This study demonstrated a statistically significant amelioration in both the subjective and objective symptoms in OLP patients treated with vitamin D supplements with or without psychological counseling apart from standard steroid therapy (Table 1).

Similar treatment outcomes were also reported by Nazeer et al. (2020) [45]. Serum vitamin D levels of all the enrolled OLP patients were evaluated., and serum vitamin D level > 30 ng/mL was considered normal. Patients with serum vitamin D levels in the range of 15–20 ng/mL, and < 15 ng/mL were considered as having moderate and severe vitamin D deficiency, respectively. Based on the serum vitamin D levels and history of stress, the patients into divided into three groups. The study concluded that vitamin D plays an important role in the management of OLP lesions. Hence, all OLP patients should be evaluated for vitamin D serum levels for improved clinical outcomes (Table 1).

Given the high incidence of OLP among post-menopausal women, a study conducted in Egypt [46], evaluated vitamin D supplementation as adjunctive therapy to topical corticosteroids in the treatment of OLP. A total of 30 post-menopausal women in the age range of 45–65 years, with an OLP diagnosis and serum 25 hydroxyvitamin D levels < 30 ng/mL were randomly allocated into one of two groups (15 patients each) as follows: control group I, received topical cortisone alone; and the intervention group II was managed with topical cortisone along with vitamin D supplementation for one month. (Table 1). Although, statistically significant pain diminution was observed in both groups, however, the intervention group receiving vitamin D supplementation exhibited a 100% resolution in the OLP lesion size.

A randomized double-blind, placebo-controlled clinical trial by Delavarian et al. (2021) [47], randomly divided the OLP patients (with serum vitamin D levels less than 30 ng/mL) into two groups: control (placebo and standard OLP therapy) and intervention group (vitamin D and standard OLP therapy). After a 2-month follow-up every 2 weeks, the study concluded that although the reduction in the pain intensity was not very remarkable, a significant diminution in the severity of lesions was observed in the intervention group (*p* = 0.043) (Table 1)

### 3.3. Assessment of Risk of Bias

**Figure 2:**
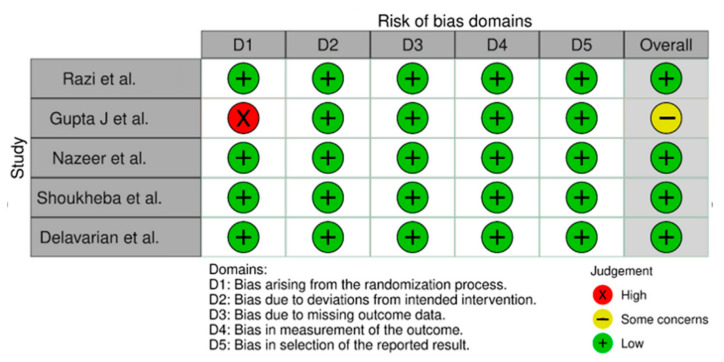
Illustrating the risk of bias domains [43,44,45,46,47].

**Figure 3:**
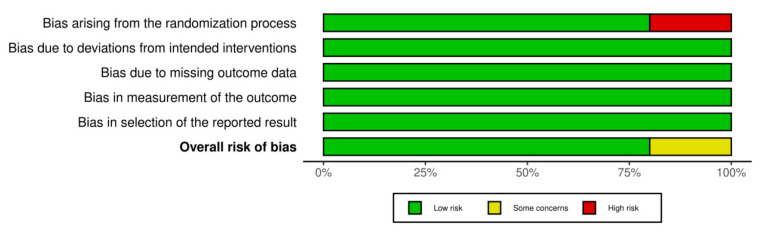
Illustrating the overall risk of bias from the included studies.

The risk of publication bias was achieved by using the R-based Robvis software package. Most of the domains showed a low risk of bias. Out of the five included studies, four studies (80%) showed a low risk of bias. Only one study (20%) showed some concerns/no information. The risk of publication bias is represented in Figure 2 and Figure 3.

## 4. Discussion

World Health Organization (WHO) defined Oral lichen planus as “a chronic auto-immune, inflammatory disorder of the skin and the oral mucosa of obscure etiology”. OLP is regarded as the most common and significant oral potentially malignant disorder [48].

The etiopathogenesis of OLP is still unclear; however, a dysregulated immune system and a multitude of predisposing risk factors may have a role in the disease etiology [4,14,17]. An immune-mediated mechanism entailing activated CD8+ T cells, acting against the basal keratinocytes, and causing alterations in the epithelial keratinization have been suggested [49]. There is an upregulated intercellular adhesion molecule-1 (ICAM-1) and cytokines associated with a helper T cell type 1 (Th1)-driven immune response. Lichen planus patients have high Th1/Th2 ratios, signifying the central role of Th1 in etiopathogenesis [50]. After the Major Histocompatibility Complex (MHC) class II antigen presentation by Langerhans cells to CD4+ T-helper cells, the stimulated cytotoxic CD8+ T lymphocytes display direct toxicity against the basal keratinocyte antigens, thus, causing basal cell layer degeneration [51].

Corticosteroids are regarded as the mainstay of OLP treatment. However, attaining complete remission and disease relapse after drug withdrawal remains a major drawback. The oral mucosa could not achieve adequate therapeutic drug levels, thus, resulting in partial resolution of the lesions. The viscoelastic nature of the oral mucosa prevents the adherence of topical paste/gels, rapidly clearing them off before getting absorbed [52]. OLP is typified by episodes of exacerbation and remissions, and a persistent protracted course. Hence, the employed long-term steroid therapy may be associated with certain local (oral candidiasis, mucosal atrophy, taste alterations, and drug hypersensitivity) and systemic adverse events (adrenocortical suppression, hyperglycemia, hypertension, psychological problems, weight gain, osteoporosis). Additionally, specific conditions such as pregnant and lactating females, herpetic infections, hypertension, diabetes mellitus, tuberculosis, glaucoma, and Human immunodeficiency virus (HIV) infection contraindicate systemic corticosteroid therapy. Hence, it is imperative to have an altered treatment protocol for OLP [53].

Vitamin D plays an essential role in the immune processes, and exhibits anti-inflammatory and antimicrobial properties, with anti-angiogenic, pro-differentiating, and antiproliferative effects [30]. 1,25-dihydroxy vitamin D (1,25(OH) 2D3)/calcitriol, the active metabolite of vitamin D, is regarded as a pleiotropic hormone and exhibits distinctive physiological activities [30]

Vitamin D modulates both the adaptive and innate immune response. The calcitriol metabolite of vitamin D interacts with nuclear vitamin D receptors (nVDR) present in immune cells (B and T lymphocytes), neutrophils, monocytes, and dendritic cells (DC) [54]. These immune and inflammatory cells also carry out the conversion of calcidiol to the active form (calcitriol) by upregulating the enzyme 1-α-hydroxylase (CYP27B1) [54,55]. Vitamin D also induces antimicrobial peptide expression like defensins β2 and β4 and cathelicidin antimicrobial peptide (CAMP) by keratinocytes, macrophages, monocytes, epithelial, pulmonary, gastric, and corneal cells, thus, augmenting chemotaxis, autophagy, phagolysosomal immune cell fusion, and strengthening the physical barrier functioning [54]. These anti-microbial properties boost the body’s defense mechanism against microbial infections [55].

Vitamin D also modulates the adaptive immune response [55,56]. Calcitriol exhibit a downregulatory effect on the cell-mediated (Th1) immune responses by suppressing the type 1 proinflammatory cytokine (such as IL-6, IL-8, IL-12, IL-17, IL-21, IFN-γ, TNF-α, and IL-9) release. However, it upregulates the humoral (Th2) response by facilitating the type 2 anti-inflammatory cytokine (such as IL-4, IL-5, and IL-10) production [54].

Interleukin (IL)-17 is the key cytokine of T-helper 17 cells implicated in the etiopathogenesis of several autoimmune and inflammatory diseases, including OLP. IL-17 not only potentiates T-lymphocyte-mediated response (facilitating the release of IL-6, IL-8, and IL-1β) but also upregulates the production of matrix metalloproteinase (including matrix metalloproteinase-9) which cleaves type IV collagen. This synergistic molecular action results in the disruption of the basement membrane and apoptosis of keratinocytes. IL-17 may further result in diminished oral bacterial heterogeneity, causing the triggering of the innate and adaptive immune response, eventually resulting in disease exacerbation. Vitamin D exerts an inhibitory effect on T-helper 17 activity by direct transcriptional inhibition of IL-17 gene expression [57].

Vitamin D also exhibits an essential role in several autoimmune disorders where antibodies have a key role. Calcitriol inhibits B cell differentiation and proliferation and promotes apoptosis [54,56]. Although vitamin D can modulate immune responses, its primary role remains anti-inflammatory [28].

OLP has an age and gender predilection, as females in the 4th-6th decade of life are commonly affected (F: M 1.5: 1) [5]. OLP has a higher predilection in peri-menopausal females (10.91%) compared to premenopausal females (0.5–2%), usually presenting with episodes of anxiety, depression, and psychological ailments. The decreased estrogen and progesterone levels may directly or indirectly (by causing depression) trigger the flare-ups of OLP lesions [58].

Estrogen augments the 1-α hydroxylase enzyme activity responsible for the active form of vitamin D; hence, the decreased estrogen levels during the menopausal phase may result in vitamin D deficiency symptoms [59]. The menopausal phase displays a substantial shift in vitamin D requirements because of the vitamin D receptor dependence on estrogen. The increased calcium demand during the menopausal transition phase also indicates vitamin D receptor loss and an augmented vitamin D demand [60].

In our systematic review, three studies included patients of both genders [44,45,47], and two studies were conducted on females (one each on peri-menopausal and post-menopausal females) [43,46].

An accurate diagnosis of OLP usually necessitates a detailed medical history, meticulous clinical and oral evaluation, coupled with histopathological examination. However, a provisional clinical diagnosis is sufficient in cases of characteristic bilaterally symmetrical, reticular oral lesions [4]. A histopathologic diagnosis not only corroborates the provisional clinical diagnosis but is also entitled to obviate cellular atypia and malignant alterations [4,61].

In our study, two studies diagnosed OLP on a clinical basis (bilateral, symmetrical interlacing Whickham’s striae, burning sensations, and intolerance to hot/spicy food) [43,46], whereas one study diagnosed OLP cases by the clinical and histopathological features based on World Health Organization (WHO) modified criteria [47]. However, two studies used the histopathological diagnosis only in doubtful cases (gingival desquamation/inconspicuous bilateral symmetrical reticular pattern) [44,45].

OLP is considered a psychosomatic disorder, with associated increased episodes of anxiety, depression, and psychic ailments in such patients. Stress attributes as the predominant predisposing factor for acute flare-ups in OLP patients [62]. Chronic stress trigger increased adrenal cortisol production and causes reduced expression of vitamin D receptors. This vicious cycle eventually results in decreased uptake/activation of vitamin D, thus affirming the possible corroboration between psychological factors, vitamin D deficiency, and OLP [63].

In our systematic review, two studies evaluated the OLP subject population based on serum vitamin D levels and history of stress. Accordingly, the study groups were treated with topical steroids and psychological counseling, topical steroids, and vitamin D supplementation, and a combination of topical steroids, vitamin D supplementation, and psychological counseling, respectively [44,45].

The workflow summarizing the use of vitamin D in OLP I is represented in Table 2.

Published studies and systematic reviews have asserted the necessity of a global comprehensive scoring system for OLP, thus, facilitating standardized outcome measures [64,65,66,67]. The scoring system by Thongprasom et al. [68] is the most frequently used in clinical trials, however, several other scoring systems for OLP have also been suggested, such as systems by Elsabagh et al. [69], Piboonniyom S-O et al. [64], Chainani-Wu N et al. [70], Escudier M et al. [71], and Kaliakatsou F et al. systems [72].

In our study, different scoring systems were used to evaluate the outcome measures. Two studies used the systems by Kaliakatsou et al. [44,45], and Thongprasom et al. [46,47], respectively. One study used the scoring systems by Silverman et al. and Escudier et al. to assess the outcome measures [43].

Meta-analysis was not carried out after the systematic review, as few studies met the inclusion criteria. These studies exhibited variable degrees of heterogeneity, either statistically or clinically, including differences in the number of participants, study designs, interventions, and outcomes.

Vitamin D deficiency is an alarming global public health concern. Globally, approximately 1 billion individuals suffer from vitamin D deficiency, and half of the population has vitamin D insufficiency [29]. Vitamin D deficiency is seen in 80% of adults in India, Pakistan, and Bangladesh [73].

Populations with high skin melanin content (reduced vitamin D synthesis in response to adequate Ultraviolet B radiation), and who practice extensive skin coverage (hijab or purdah system particularly in Middle Eastern countries) are more vulnerable to vitamin D deficiency. Inadequate sunlight exposure due to excessive sunscreen use, extended indoor and limited outside activities; inadequate dietary intake (particularly vegans); body mass index (BMI); age and gender (elderly and multiple pregnancies in females); obese/overweight and malabsorption states (celiac disease, inflammatory bowel disease or liver cirrhosis); serum calcium and parathyroid hormone levels; and medication such as steroids are several other factors that may influence vitamin D status [29,73,74].

Two studies in our systematic review were from India [44,45], and one study each was from Pakistan [43], Egypt [46], and Iran [47].

[25(OH)D]/calcidiol, the main circulating metabolite of vitamin D is present at nanomoles/liter concentrations. It provides a superior expression of vitamin D stores due to a longer half-life of 3 weeks. 1,25-dihydroxycholecalciferol [1,25(OH)2D3] is the active vitamin D metabolite with a shorter half-life (4–15 h) and is present at picomoles/liter concentrations in circulation [73,74].

High-performance liquid chromatography (HPLC), radioimmunoassay (RIA), enzyme-linked immunoassay (ELISA), and quantitative chemiluminescent immunoassay (CLIA) are the few commonly employed techniques for the clinical detection of total 25(OH)D. Liquid chromatography/tandem mass spectrometry (LC-MS/MS) is now regarded as the gold standard method for analyzing the various forms of vitamin D [30].

In our systematic review, only one study mentioned the technique used for vitamin D estimation. A study by Delavarian et al. [47] measured serum vitamin D levels by the enzyme-linked immunosorbent assay (ELISA) method.

The definition of sufficient serum vitamin D levels remains contentious with conflicting suggestions from different advisory councils. The US National Academy of Medicine (formerly Institute of Medicine) suggests serum vitamin D levels of 20 ng/mL and above (50 nmol/L or above) as sufficient, however, the US Endocrine Society and International Osteoporosis Foundation guidelines consider levels of 21–29 ng/mL as “insufficient” and <20 ng/mL as “deficient” [27,75,76]. Additionally, the appropriate vitamin D levels may even differ between individuals or in the same person with different cellular activities (such as immunoregulatory effects or calcium–phosphorus homeostasis) [74].

One of the first studies to ascertain the efficacy of vitamin D supplementation as a treatment modality was done by Razi et al. [43]. Their outcome parameters were primarily based on the VAS scale assessment for pain and the reduction in the lesion size after 1 and 4 weeks of treatment, respectively. In the subsequent year, Gupta J et al. [44] did a similar study to further establish the role of vitamin D supplementation in lichen planus. They divided their cohort into three groups based on the levels of vitamin D deficiency. They also assessed the outcome based on the VAS scale for pain and reduction in the size of the lesion. Similar to Gupta et al., Nazeer et al. [45] also conducted a study with the same outcome parameters, however with a much larger sample size of 450 patients. Shoukheba et al. [46] conducted a randomized controlled trial and observed a significant reduction in pain scores (VAS) and size of the lesion compared to the baseline in both groups. Another similar randomized double-blind, placebo-controlled clinical trial was conducted by Delavarian et al. [47]. The reduction in pain (VAS) scale and lesion size were evaluated every two weeks for a total of eight weeks.

Irritant contact dermatitis and hypercalcemia are the commonly reported adverse effects of vitamin D analogs [38]. However, no such adverse events were documented in our study.

Recent research has documented that OLP patients may present with dysbiosis [77,78,79]. Paraprobiotics and postbiotics play a proactive role in the maintenance of eubiosis. Paraprobiotics are a novel ancillary treatment strategy for periodontal disease. Paraprobiotics not only serve as an efficacious regimen for the domiciliary maintenance of oral health but also assess the cellular and inflammatory variables due to their immunomodulatory effects [80]. Another study used a postbiotic-based gel containing lactoferrin and aloe barbadensis leaf juice powder as a treatment regimen for periodontitis [81].

These recent developments highlight the importance of newly introduced therapies in various dental fields. However, future research is needed to improve current knowledge about the treatment modalities in OLP.

One of the limitations of our review was that the literature search was carried out on only two search engines. The diagnostic criteria employed for OLP also varied in the included studies. Additionally, none of the included studies, followed up with the patients to complete OLP remission, and if vitamin D supplementation was prescribed for long-term use or not. Among the few included studies, there were variable degrees of heterogeneity, either statistically or clinically, including differences in the number of participants, study designs, interventions, and outcomes, thus, posing a challenge to perform a meta-analysis. At the outcome level, the presence of confounding factors such as ethnicity, demographical differences, the technique employed to evaluate serum vitamin D levels, serum calcium, and parathyroid hormone levels, and body mass index (BMI) also added to the limitations of the study. Only one of the included studies mentioned the technique employed to evaluate serum vitamin D levels (Delavarian et al. [47] study measured serum vitamin D levels by the enzyme-linked immunosorbent assay (ELISA) method). The technique used for the estimation of serum vitamin D levels may further affect the disease severity and its clinical outcome [43]. Another limitation, as asserted previously is the lack of a global comprehensive scoring system for OLP, thus facilitating standardized outcome measures.

## 5. Conclusions

Despite meticulous attempts to ascertain a definitive therapeutic protocol, there is no conclusive therapeutic regimen in OLP owing to its obscure etiopathogenesis. Our study results demonstrated a significant amelioration in the OLP symptoms in patients who were given vitamin D supplements as an adjuvant to the conventional steroid therapy and or placebo. However, well-designed prospective clinical trials with large sample sizes need to be carried out to establish and corroborate the therapeutic role of vitamin D in OLP.

## Figures and Tables

**Table 1:** Detailed representation of the included studies.

Sn	Author (s)/Year/Country	Type of Study	Age/Sex/Follow Up	Sample Size	Oral Lichen Planus (OLP) Diagnosis	Treatment Plan				Test of Significance	Outcome					Conclusions
1.	Razi et al., 2018 [43] Pakistan	Randomized controlled Clinical Trial	35–45 years/Peri-menopausal females; 4 weeks follow up	100	Clinical diagnosis	OLP patients with vitamin D serum levels below 30 ng/mL were divided into 2 groups:				Paired Sample T-test	Visual analog scale (VAS) score					Patients receiving standard therapy + Vitamin D supplementation (Group II) exhibited amelioration in the clinical appearance of the lesion between week 1 and week 4.
											Group		Week 1		Week 4	
											I		5.04 ± 2.20		1.80 ± 0.40	
											II		1.80 ± 0.40		0.80 ± 0.40	
						Group	Vitamin D supplement		Steroids		Size of Lesion					
											Group		Week 1		Week 4	
						I			***		I		1.80 ± 1.40		1.48 ± 0.74	
						II	***		***		II		1.80 ± 1.40		0.80 ± 0.40	
2.	Gupta J et al., 2019 [44] India	Observational study	All age groups/Both genders, 12 weeks follow up	106	Clinical Diagnosis based on typical bilateral white interlacing Whickham’s striae, burning sensations and intolerance to spices. However, doubtful cases (Gingival desquamation/inconspicuous reticular pattern) were biopsied for a confirmatory OLP diagnosis.	OLP patients were divided into 3 groups based on Vitamin D levels, and history of Stress.				Fischer’s Exact test	VAS (Pain score)					(1) Patients treated with vitamin D supplementation (Group II and III) reported statistically significant amelioration in OLP symptoms. (2) Patients treated with vitamin D supplements and psychological Counseling (Group III) reported a marked diminution in the burning sensations.
						Group	Counseling	Vit D	Steroids		Group		0–4		>4	
											I		66.70%		33.30%	
											II		73.90%		26.10%	
											III		93.30%		6.70%	
						I	***		***		Size of Lesion					
						II		***	***		Group		0–2		3–5	
											I		46.70%		53.30%	
						III	***	***	***		II		86.90%		13.10%	
											III		86.70%		13.30%	
3	Nazeer et al., 2020 [45] India	Observational study	35–45 years/Both genders; 4 and 15 weeks follow up	450	Clinical Diagnosis based on typical bilateral white interlacing Whickham’s striae, burning sensations and intolerance to spices. However, doubtful cases (Gingival desquamation/inconspicuous reticular pattern) were biopsied for a confirmatory OLP diagnosis.	OLP patients were divided into 3 groups based on their serum Vitamin D levels and history of stress.				ANOVA test	VAS (Pain score)					(1) Patients treated with vitamin D supplementation reported a statistically significant amelioration in subjective symptoms (Group I and II).
						Group	Counseling	Vit D	Steroids		Group		0–4		>4	
											I		54.70%		45.30%	
											II		64.70%		35.30%	
						I	***	***	***		III		33.30%		66.70%	
											Size of Lesion					
						II		***	***		Group		0–2		3–5	
											I		86.70%		13.30%	
						III			***		II		56.70%		43.30%	
											III		46.70%		53.30%	
4	Shoukheba et al., 2020 [46] Egypt	Randomized controlled Clinical Trial	45–65 years/Post-menopausal females; 2,4,6 weeks follow up	30	Clinical diagnosis	OLP patients with serum Vitamin D levels below 30 ng/mL were randomly divided into 2 groups.				Paired Sample T Test	VAS (Pain score)					(1) A statistically significant reduction in pain scores (VAS) compared to the baseline data was observed with both groups. (2) At 6 weeks of follow up Group II receiving Vitamin D supplementation showed a 100% reduction in lesion size.
											Group	Week 2	Week 4		Week 6	
											I	2.8 ± 0.67	1.73 ± 0.70		2.8 ± 0.63	
											II	2.13 ± 0.91	1.33 ± 0.70		1.86 ± 0.51	
											Group	Week 2	Week 4		Week 6	
						Group	Vitamin D supplement		Steroids		Size of Lesion					
											Group		0–2		3–5	
						I			***		I		46%		54%	
						II	***		***		II		100%		0%	
		
5.	Delavarian et al., 2021 [47] Iran	Randomized double-blind, placebo-controlled clinical trial	22–70 years old/both genders (mostly females)	28	Clinical and histopathological Diagnosis based on World Health Organization (WHO) modified criteria.	Based on OLP diagnosis and vitamin D levels less than 30 ng/mL, 28 patients were divided into 2 groups. Group I (Intervention group; n = 13) and Group II (Control group; n = 15)				Paired Sample T Test	VAS (Pain score)					A significant decrease in the severity of lesions was observed in the intervention group (*p* = 0.043).
											Group	Week 2	Week 4	Week 6	Week 8	
											I	7.38 ± 3.25	4.13 ± 2.64	2.75 ± 2.43	2.13 ± 2.33	
											II	1.21 ± 1.67	0.93 ± 1.32	1.29 ± 2.16	1.64 ± 2.31	
						Group	Vitamin D supplement	Steroids	Lactose		Size of Lesion					
											Group	Week 2	Week 4	Week 6	Week 8	
						I	***	***			I	3.63 ± 0.92	3.63 ± 0.74	3.38 ± 0.52	3.50 ± 0.93	
						II		***	***		II	3.14 ± 0.54	3.07 ± 0.92	3.07 ± 0.62	3.14 ± 0.86	

Note: *** denotes the prescribed treatment in different groups.

**Table 2:** Workflow of vitamin D in Oral Lichen Planus.

	Salient Property	Mechanism
1.	Anti-inflammatory and Immunomodulatory	Vitamin D also induces antimicrobial peptide expression like defensins β2 and β4 and cathelicidin antimicrobial peptide (CAMP) by keratinocytes, macrophages, monocytes, epithelial, pulmonary, gastric, and corneal cells, thus, augmenting chemotaxis, autophagy, phagolysosomal immune cell fusion, and strengthening the physical barrier functioning. These anti-microbial properties boost the body’s defense mechanism against microbial infections. Vitamin D modulates both the adaptive and innate immune response. The calcitriol metabolite of vitamin D interacts with nuclear vitamin D receptors (nVDR) present on immune cells (B and T lymphocytes), neutrophils, monocytes, and dendritic cells (DC). Calcitriol exhibit a downregulatory effect on the cell-mediated (Th1) immune responses by suppressing the release of type 1 proinflammatory cytokines (such as IL-6, IL-8, IL-12, IL-17, IL-21, IFN-γ, TNF-α, and IL-9). However, it upregulates the humoral (Th2) response by facilitating the production of type 2 anti-inflammatory cytokines (such as IL-4, IL-5, and IL-10).
2.	Keratinocyte proliferation and differentiation	Vitamin D has a regulatory effect on keratinocyte proliferation and differentiation. Calcitriol inhibits B cell differentiation and proliferation and promotes apoptosis. Vitamin D/analogs may facilitate the restoration of the normal epidermal cytokeratin profile, thus, further attributing to its therapeutic potential in lichen planus.
3.	Adrenal cortisol regulation	Increased episodes of anxiety, depression, and psychic ailments have been associated with OLP patients. Chronic stress attributed as the predominant predisposing factor for acute flare-ups in OLP triggers increased adrenal cortisol production and causes reduced expression of vitamin D receptors. This vicious cycle eventually results in decreased uptake/activation of vitamin D, thus, affirming the possible corroboration between psychological factors, vitamin D deficiency, and OLP.

## Data Availability

Not applicable.
